# Patient safety culture in a university hospital emergency department in Switzerland – a survey study

**DOI:** 10.3205/zma001222

**Published:** 2019-03-15

**Authors:** Meret E. Ricklin, Felice Hess, Wolf E. Hautz

**Affiliations:** 1 Inselspital Bern, Universitäres Notfallzentrum, Bern, Switzerland

**Keywords:** patient safety, emergency department, medical errors, survey

## Abstract

**Aim of the study: **Poor safety culture, bad teamwork, non-functional inter-departmental working relationships and increased cognitive demands are associated with higher amounts of adverse events in hospitals. To improve patient safety, one of the first steps is to assess safety culture among health care providers in an institution. Considering the sparsity of studies addressing patient safety culture in Europe and Switzerland, the aim of the present study was to assess patient safety culture in the emergency department of a University Hospital in Switzerland.

**Methods: **We employed the Hospital Survey On Patient Safety Culture, developed by the U.S. Agency for Healthcare Research and Quality. 140 questionnaires were distributed to nurses and physicians. Two weeks after the first questionnaire, we performed a sensitization campaign addressed to health care providers, and then repeated the survey. We calculated composite scores for each question category and percentages of positive responses for each dimension. For group comparisons such as possible differences relating to education and duration of employment and to compare results of the first and second survey we used T-tests. The results were compared to other published surveys outside of Switzerland.

**Results: **Particularly positive assessments were found for the categories “nonpunitive response to errors”, “teamwork within units”, “supervisor/manager expectations and actions promoting patient safety” and, compared to other hospitals, also “staffing”. The lowest average percent positive responses were found in the categories “frequencies of reported event”, “teamwork across units” and “handoffs and transitions”. Nurses and health care personnel with a longer employment history had an overall more negative assessment of patient safety culture, when compared to physicians and personnel with a shorter duration of employment, respectively.

**Conclusions: **The present study has identified strengths and potential weaknesses in the safety culture of a large university hospital emergency department in Switzerland. The results provide opportunities for improvement of patient safety in particular in the reporting of adverse events, in interaction across units and patient transitions. Furthermore, as we employed a standardized self-assessment tool similar to previously published studies, the work contributes to the establishment of a benchmark for hospital safety culture at the national, European and international level.

## Zusammenfassung

**Ziel der Studie: **Schlechte Sicherheitskultur, schlecht abgestimmte Zusammenarbeit, nicht-funktionierende Übergänge zwischen verschiedenen Abteilungen und verstärkte kognitive Herausforderungen gehen einher mit häufigeren fehlerhaften Behandlungen in Spitälern. Einer der ersten Schritte um die Patientensicherheit zu steigern, ist die Sicherheitskultur der Gesundheitsfachleute innerhalb einer Institution zu verbessern. Wegen der spärlich vorhandenen Studien über Patientensicherheit in Europa und der Schweiz war das Ziel dieser Studie die Patientensicherheitskultur in einem universitären Notfallzentrum in der Schweiz zu untersuchen.

**Methoden: **Wir haben die deutsche Übersetzung (PASKI) des Fragenbogens zur Patientensicherheitskultur „Hospital Survey On Patient Safety Culture“, den die amerikanische „Agency for Healthcare Research and Quality“ etabliert hat, benutzt. 140 Fragebögen wurden unter Pflege- und Arztpersonal verteilt. Zwei Wochen nach der Verteilung der ersten Fragebögen haben wir unter den Gesundheitsfachpersonen eine Informationskampagne gestartet und danach die Umfrage wiederholt. Wir haben die Werte für jede Fragen-Gruppe berechnet und auch die prozentualen positiven Anteile der einzelnen Fragen.

Zum Vergleich von Gruppen, wie zum Beispiel Unterschiede zwischen verschiedenen Ausbildungen, oder unterschiedlich langen Anstellungsverhältnissen und auch zum Vergleich des erstens und des zweiten Fragebogens habend wir einen T-Test benutzt. Die Resultate haben wir mit anderen, publizierten Daten von ausserhalb der Schweiz verglichen.

**Resultate: **In den Gruppen „nicht strafende Reaktion auf Fehler“, „Zusammenarbeit innerhalb der Abteilung“, „Erwartungen von Vorgesetzten und Massnahmen, die Patientensicherheit fördern“ und vor allem im Vergleich zu anderen Spitälern „Personalbestand“ wurden vor allem positive Antworten gefunden. Die tiefsten Antworten wurden bei den Kategorien „Anzahl gemeldeter unerwünschter Ereignisse“, „Abteilungsübergreifende Zusammenarbeit“ und „Übergabe und Schichtwechsel“ gefunden.

Pflegende und Personen, mit bereits längerem Anstellungsverhältnis gaben eine schlechtere Beurteilung zur Patientensicherheit ab im Vergleich zu Arztpersonal und Gesundheitsfachleuten, die noch nicht so lange hier angestellt waren.

**Schlussfolgerungen: **Diese Studie hat einige Stärken und potentielle Schwächen in der Patientensicherheitskultur in einem grossen universitären Notfallzentrum in der Schweiz aufgedeckt. Die Resultate bieten die Möglichkeit, die Patientensicherheitskultur zu verbessern, insbesondere im Melden von unerwünschten Ereignissen, der Interaktion zwischen Abteilungen und der Patientenübergabe. Da wir einen standardisierten Fragebogen benutzt haben, den die Befragten selber ausfüllen, wie er in anderen Studien auch schon benutzt wurde, hilft diese Studie eine Benchmark für eine nationale, europäische und internationale Patientensicherheitskultur in Spitälern zu etablieren.

## Introduction

In 1999 the Institute of Medicine (IOM) from the National Academy of Science, Engineering, and Medicine (USA) released a report about fatal medical errors, estimating an incidence rate of 44.000-98.000 deaths per year in the U.S. [1]. In North America, this provoked an intense discussion followed by many interventions as well as further studies. The definition of a medical error was “a failure of a planned action to be completed as intended”, or “the use of a wrong action to achieve an aim”. This IOM study reported that the highest error rates resulting in serious consequences were most frequently occurring in intensive care units, operating rooms, and emergency departments (ED) [1]. A recent retrospective study by our group confirmed the suspicion that adverse events may be frequent and relevant in emergency medicine. When retrospectively comparing the interpretation of all radiographic images taken at the ED of Inselspital Bern to the delayed but definitive reading by a senior radiologist, we found a discrepancy rate of more than 20%. The primary interpretations were extracted from the EDs discharge letter and stem from the joint reading of the images of a junior radiologist on duty and the attending emergency physicians. The study thus compares the EDs radiology based diagnoses to a gold standard. When compared to this gold standard of a certified radiologist, one third of the differences identified were judged clinically relevant by two independent expert raters [2]. 

WHO estimates that in Europe 10% of hospitalized patients suffer from preventable harm, and a recent study from Switzerland reported that 5.9% of hospitalized patients suffered from health care associated infections [3], [4]. Other studies reported an incidence of adverse events of 7.5%, the majority of which was considered preventable [5], [6]. 

Suggested strategies for the reduction of medical errors included an enhancement of patient safety culture, the introduction of a reporting system enabling to learn from errors without blame, raising performance standards and enduring safe practices at the delivery level [1]. Importantly, several studies indicate that adverse events are impacted by patient safety culture [7], [8], [9]. Patient safety culture has been defined as the “values shared among organization members about what is important, their beliefs about how things operate in the organization, and the interaction of these with work unit and organizational structures and systems, which together produce behavioral norms in the organization that promote safety” [7], [8], [9], [10]. To improve patient safety, one of the first steps is to assess safety culture among health care heath care professionals (HCP) in an institution [11]. To this end, the safety climate reported by the professionals has been previously employed [12].

Considering the sparsity of studies addressing patient safety culture in Europe in general and Switzerland specifically, the aim of the study was to assess patient safety culture among HCP in the ED of the University Hospital of Bern. We employed a well-established survey developed by the U.S. Agency for Healthcare Research and Quality [13]. As of March 2017, this survey had been used in 71 countries in 32 translations [13], [14], [15], [16], [17], [18].We furthermore tested the impact of an information campaign about patient safety on the results of the survey.

## Materials and Methods

### Surveys on patient safety culture

For the present self reported, uncontrolled before and after study, the ED of the University Hospital of Bern “Universitäres Notfallzentrum” (UNZ) was selected, a large, level one and university affiliated ED that sees around 45.000 patients annually [19]. To assess patient safety culture among all HCP working in the emergency department we used the Hospital Survey On Patient Safety Culture (HSOPSC) questionnaire, that was translated into German and validated for Switzerland (named PaSKI) [13], [16], [20]. This questionnaire is a well established tool, developed by the U.S. Agency for Healthcare Research and Quality [13]. As of March 2017, this survey had been used in 71 countries in 32 translations [13], [14], [15], [16], [17], [18]. Questionnaires were distributed among all nurses and physicians working in the ED. People on longer absences and undergraduate students were excluded, as the later did not stay long enough to cover the whole length of the study. We distributed the survey in the beginning of May 2017 approaching each HCP personally. Completed questionnaires were collected and digitized. 

Two weeks after the first questionnaire we performed an intervention to sensitize HCP. To this end, several activities occurred between mid-May to mid-June 2017. The campaign included information about the importance of patient safety, medical errors and the blame-free possibility of reporting adverse events. The HCPs were sensitized by several short talks at shift-changes, publication of information in the internal newsletter with the support and signature of both the head of the emergency department and the head of nursing, and by distribution of an information sheet among all HCP. In July 2017, the second survey was administered. Again, 140 of the identical surveys were distributed personally among the same HCP. HCP were asked to fill in a code on each survey to match the second round to the first. Surveys were again collected and digitized.

#### Evaluation of surveys and statistical analyses

Surveys with less than 30% of answers were excluded. The results of negatively worded questions were reverse coded for the analyses of the individual items. We calculated composite scores for each question category (termed composite) as suggested by Sorra and Nieva [21] and as described by the agency for Healthcare Research and Quality (AHRQ, Rockville, MD, USA) by calculating the average of corresponding items [13]. Each composite was composed of three to four items. For each question, five answers were possible: from “never” to “always” or from “I strongly disagree” to “I strongly agree”, respectively. We also calculated percentages of positive responses for each dimension by dividing the number of positive responses on corresponding items by the number of non-missing answers in the composites. The two highest positive answers were pooled as suggested by the AHRQ to make results comparable to previously published data. Changes of 5% or more are considered to be potentially relevant [13], [21]. In general, higher scores represent a better patient safety culture.

Descriptive statistics for each item and dimension (mean and standard deviation (SD)) were evaluated. 

Analyses were conducted using SPSS V.22 (IBM, Armonk, New York, USA) and Microsoft Excel (Redmond, WA, USA). All survey data were further analyzed regarding their statistical distribution and tested for normality by means of the Shapiro Wilks test. Because no distribution was significantly different from normality we employed T-tests to test for differences between groups. Groups were formed based on baseline characteristics such as age (older versus younger) or profession (physician versus non-physician HCP). Furthermore, our surveys (before and after) were analyzed separately using composites and were compared to each other by T-tests.

Our findings are descriptively compared to the U.S. American Database of 2016 [15], and two studies from Germany and Sweden, respectively [22], [23]. These were selected as they contained enough detailed information for descriptive comparisons to our data. However, statistically comparing our data against both studies is not possible because neither provides raw data.

## Results

### Overview on returned questionnaires from the UNZ

Only one questionnaire had to be excluded, due to too many missing answers. The overall return rate in the first round was 101 of 140 (72%) and in the second round 53 of 139 (38%). For detailed demographic data see table 1 (Tab. 1). 20% of physicians and 10% of nurses were male. More than half of the participants were younger than 40 years old (58%) and 72% of the responders worked 80% (33.6h/week) or more. Almost two thirds of the HCP had worked at the UNZ for less than 5 years at the time of the survey. 

Attachment 1 shows the positive response for each question and question category (composite). Before analysis, a reverse coding for negatively worded questions was performed. The three composites with the highest average percent positive responses were “nonpunitive response to errors” with 78.7% positive answers, “teamwork within units” with 70.1% positive answers and “supervisor/manager expectations and actions promoting patient safety” with 67.9%. The three composites with the lowest average percent positive responses were “frequency of events reported” with 37.8% of positive answers, “teamwork across units” with 46.88% and “handoffs and transitions” with 47.4% positive responses. 

#### Comparison of results with other hospitals

**Figure 1:**
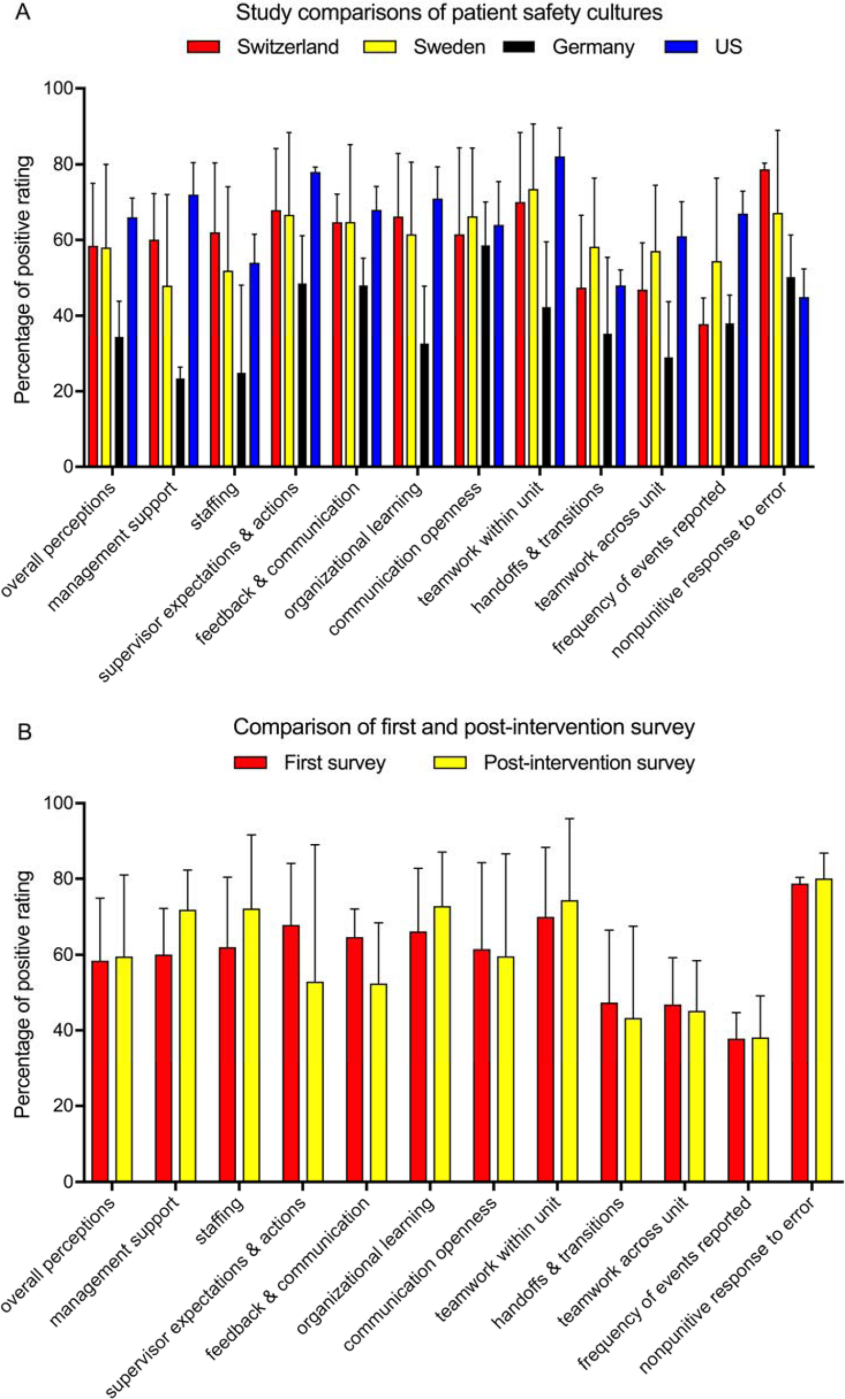
Overview of the survey results. In (A), the results for each composite from the UNZ was compared to published data from Sweden, Germany and the US [15], [23], [24]. Averages and SD calculated from the different questions of the composites are shown. In (B), a comparison of first and post-intervention survey performed at the UNZ is shown.

With the aim to enable a better interpretation of the results, we made a comparison with previous reports from Sweden, Germany and the United States (US), which used the identical questionnaire to assess patient safety culture in hospitals [15], [22], [23]. Overall, the responses we obtained appear to be similar to those from a Swedish ED, but more positive than those from Germany, and in most cases less positive than those from the US (see figure 1 (Fig. 1), Point A). Although a statistical analysis is not possible due to lacking access to the raw data, these comparisons indicate that the strength of the UNZ was in the area of “staffing” and “nonpunitive response to error“ (see figure 1 (Fig. 1), Point A). Interestingly, despite this, the “frequency of reported errors” was amongst the lowest at the UNZ, while the US survey had the highest “frequency of reported errors” despite having the lowest “nonpunitive response to error”. This finding exemplary points to possible consequences to be drawn: At the UNZ, a systematic incident reporting system exists. However, two third of the responders (65; 66.3%) indicated not to have reported any incidence within the last six months, 27 indicated to have reported one to two and six reported three to five incidents in the last six months. Altogether, these data would indicate that the “frequency of reported errors”, “teamwork across units” and “handoffs and transitions” would be the most important areas for improvement in the investigated ED. 

#### Parameters impacting the overall perception of patient safety 

Interestingly, the overall perception of patient safety, which was assessed in the survey by a subjective score for patient safety given by each HCP, was higher by physicians (78%) than by nurses (60%). Nevertheless, none of the responders rated it poor or failing (see table 2 (Tab. 2) for details). Older responders graded communication openness as more positive than younger ones (p=0.037). Our data also showed that employees with a lower professional degree stayed longer in the ED (p=0.033). Interestingly, this group also reported more adverse events (p=0.0001) compared to HCP with a higher professional degree. When compared to their colleagues working for a shorter time period in the ER, the staff working for longer in the department also rated staffing to be less good than their colleagues (p=0.001) and also felt handoffs and transitions were less positive (p=0.001).

Physicians thought they would do more for patient safety than do nurses (p=0.001) and they also rated the communication openness (p=0.0001) and the team work across units (p=0.013) to be better than their nursing colleagues. 

#### Impact of intervention on the responses to the questionnaire

After the educational intervention, a second identical survey was distributed to assess, if patient safety culture improved. Using a t-test we did not find any significant differences between the two time points. After the intervention, the positive response for “management support”, “staffing” and “organizational learning” increased by 11.8%, 10.2% and 6.7%, respectively. Nevertheless, positive perception of “supervisor expectations & actions” and “feedback & communication” decreased by 15% and 12% (see figure 1 (Fig. 1), Point B). Although these differences were not statistically significant, the HSOPSC guidelines state that they may be considered as potentially relevant, because they exceed 5% [13], [21].

## Discussion

Several studies indicate that a health care system can be improved by a paradigm shift in which errors are not primarily viewed as the result of individual failure but rather as opportunities to improve a system [20], [24]. High risk industries have achieved an excellent safety performance from which the health care industry can learn [25]. In fact, independent studies have found that poor safety culture, poor team work, poor inter-departmental working relationships and increased cognitive demands such as “taking difficult decisions” and “coping with many tasks at the same time” have been identified to be associated with more adverse events independently [7]. 

To establish a positive patient safety climate, the first step is to assess the present situation. For this purpose, self-reporting instruments like the one used in our study are commonly used. Although the weaknesses of self-reporting and uncontrolled before and after studies are widely recognized, the assessment of patient safety culture represents an important starting point for raising awareness to the problem, identifying and evaluating possible interventions and establishing internal and external benchmarking [1], [5], [6], [26], [27]. A selective intervention in e.g. only half of the department would not have been possible for practical reasons, as people are not separated but work and talk together. However, it is important to employ a standardized and identical tool in order to be able to compare the results obtained with those found in other hospitals. In general, scores in our sample were lower than in U.S. hospitals with the exception of “staffing” and “nonpunitive errors”. The reason for the higher value for “staffing” could be that the investigated ED indeed is in a comfortable situation with respect to trained human resources compared to the non-Swiss hospitals. Along the same line, it is possible that a lack of staff in the German EDs was an important factor contributing to the overall more negative responses found there [23]. The fact that “nonpunitive response to errors” received a very high percentage of positive answers (78.7%) is a compliment to the UNZ’s leadership concerning their error-culture. In fact, the most important factor considered to improve patient safety culture has been reported to be an organizational culture that encourages reporting without blaming, and one which promotes communication between health care practitioners [28]. 

The high rating for “nonpunitive response to errors” combined with the relatively low “frequency of events reported“ is thus rather surprising. The first should stimulate the latter. Nevertheless, the fact that a reverse response to these composites was found for U.S. hospitals indicates that the responses to “frequency of events reported” is likely to be strongly influenced by factors other than nonpunitive responses to a reported error. In particular, this could be a lack of awareness for the positive effects of reporting mistakes on the long-term improvement of patient safety. Since the responses to our second survey after the intervention were very similar to before, this could indicate a generally good awareness for patient safety but not for the importance of reporting adverse events. Additionally time between the intervention and the second survey may have been too short to expect much effect on reporting. To shed light on this question, a study assessing the incidents of adverse events retrospectively in the investigated ED is ongoing. 

In conclusion, the present study using the HSOPSC (PaSKI) questionnaire has identified strengths and potential weaknesses in the safety culture of a large university hospital ED in Switzerland. The results provide opportunities for improvement of patient safety but also warrant further research in this topic. For the UNZ areas important for improving are the reporting of adverse events, handoffs and transition and the teamwork across units. This is supported by several studies done in the UNZ in this field [29] [30], [31]. Furthermore, with more data available based on standardized self-assessments a benchmark for a hospitals safety culture may be established. These studies should be accompanied by research measuring objective error rates, such as e.g. diagnostic error [30], [32], [33]. We therefore recommend that future studies should be performed in other Swiss and European hospitals. Such studies are especially critical to the field of emergency care [33]. 

## Acknowledgements

The PASKI questionnaire (German translated and validated version of the HSOPSC) was kindly provided by Professor Tanja Manser (Director FHNW School of Applied Psychology, Olten, Switzerland). We thank Artur Summerfield for critically reading the manuscript. We further gratefully acknowledge the support of Aristomenis Exadaktylos and Petra Fuchs, director and director of nursing at UNZ and all participants of the survey.

## Author’s contribution

MER conceived the idea, designed the study, analyzed data, and wrote the manuscript. FH was involved in the study design and data acquisition. 

WEH conceived the idea, designed the study, analyzed data, and critically read the manuscript.

## Competing interests

WEH received speakers honorarium from AO foundation Zürich and sponsoring for a conference he organizes from Mundipharma Medical Basel. All other authors declare that they have no competing interests to report. 

## Supplementary Material

Positive response rate of each item (the highest two positive answers pooled) of the first survey. Negatively worded questions were reverse coded. High scores stand for positive answers.

## Figures and Tables

**Table 1:**
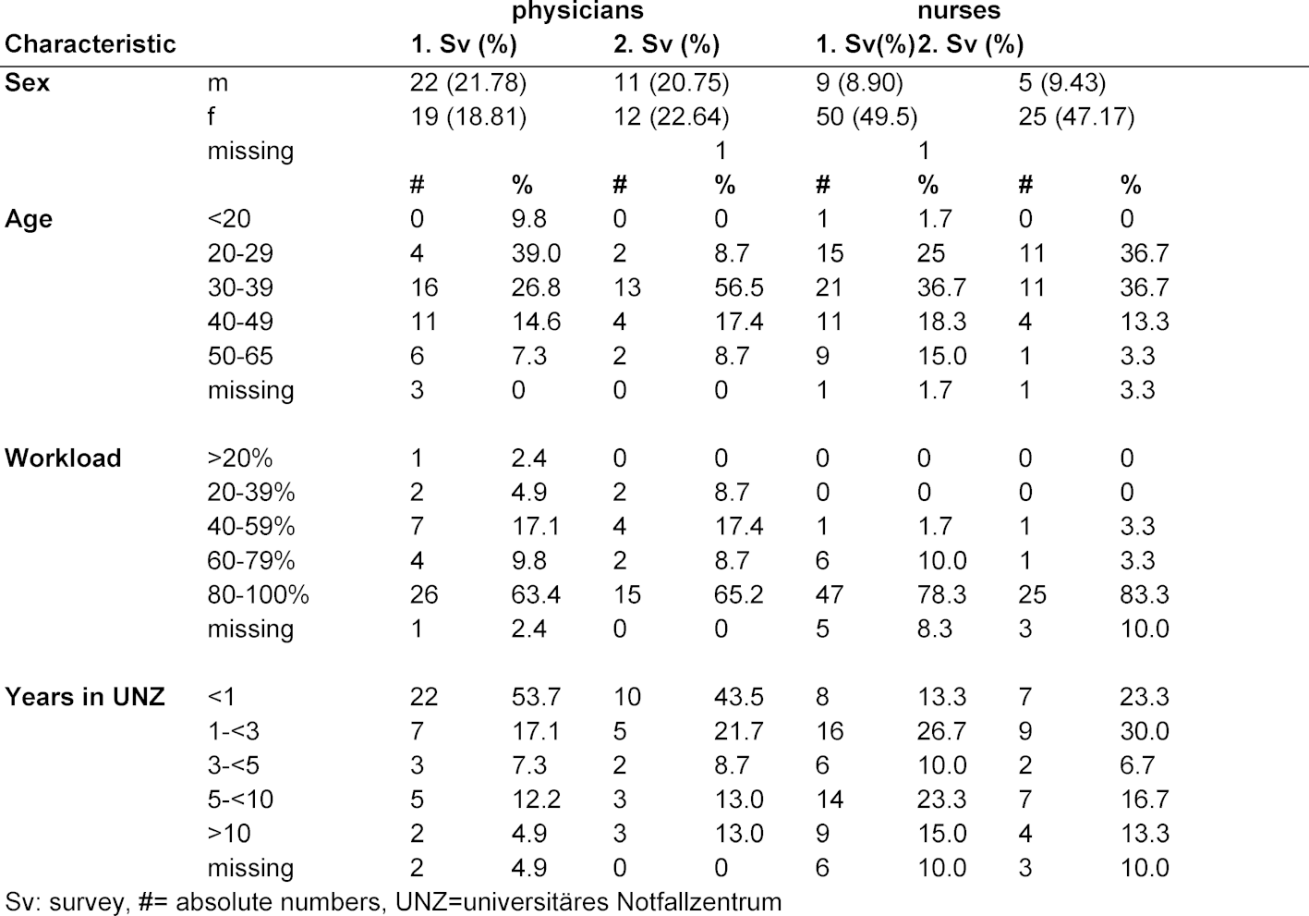
Demographic characteristics of the responders

**Table 2:**
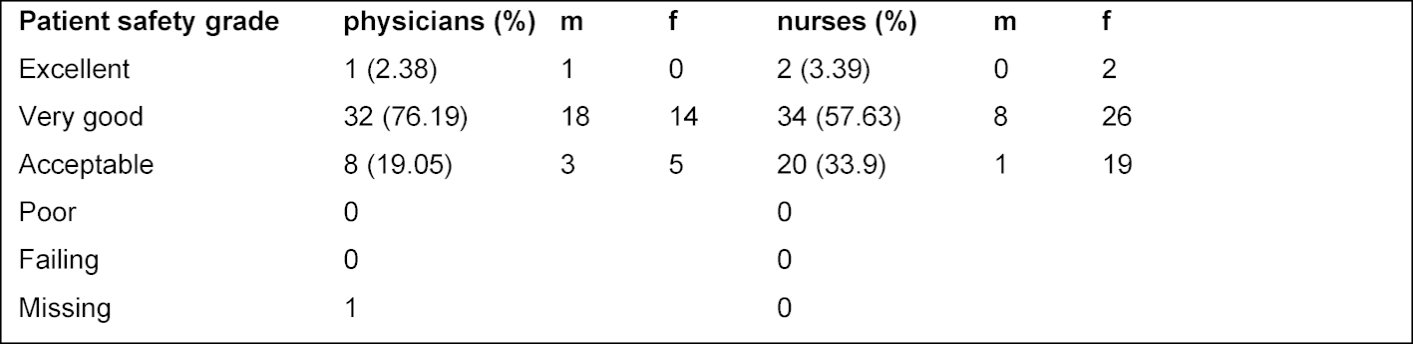
Patient safety grade based on a subjective score given by each HCP for patient safety in the hospital. Comparisons of numbers of ratings by nurses, physicians and sex for the first round of the survey are shown.
