# Supplementary material for: Patient safety culture in a university hospital emergency department in Switzerland – a survey study
Source: GMS J Med Educ. 2019 Mar 15;36(2):Doc14. doi: 10.3205/zma001222 (PMC6446463; doi:10.3205/zma001222)
Supplement: Positive response rate of each item (the highest two positive answers pooled) of the first survey. Negatively worded questions were reverse coded. High scores stand for positive answers. [file JME-36-2-14-s-001.pdf]

Composites and items	Mean % (SD)	Mean (SD)
**Teamwork within units**	**70.07 (18.24)**	
A1. People support one another in this unit.	81.63	4.15 (0.71)
A3: When a lot if work needs to be done quickly, we work together as a team to get the work done.	81.63	3.99 (0.72)
A4: In this unit, people treat each other with respect.	73.74	3.8 (0.65)
A11: When one area in this unit gets really busy, others help out.	43.29	3.24 (1.02)
**Supervisor/Manager expectations & actions promoting patient safety.**	**67.94 (16.16)**	
B1: My supervisor/manager says a good word when he/she sees a job done according to established patient safety procedures.	54.26	3.51 (0.96)
B2: My supervisor/manager seriously considers staff suggestions for improving patient safety .	80.85	3.88 (0.67)
B3: Whenever pressure builds up, my supervisor/manager wants us to work faster, even if it means taking shortcuts.	53.68	3.38 (0.92)
B4: My supervisor/manager overlooks patient safety problems that happen over and over.	82.98	3.95 (0.64)
**Organizational learning – continuous improvement**	**66.20 (16.54)**	
A6: We are actively doing things to improve patient safety.	84.69	4.00 (0.59)
A9: Mistakes have led to positive changes here.	61.11	3.60 (0.71)
A13: After we make changes to improve patient safety, we evaluate their effectiveness.	52.81	3.54 (0.72)
**Management support for patient safety**	**60.12 (12.14)**	
F1: Hospital management provides a work climate that promotes patient safety.	62.10	3.65 (0.66)
F8: The actions of hospital management show that patient safety is a top priority.	71.15	3.36 (0.85)
F9: Hospital management seems interested in patient safety only after an adverse event happens.	47.12	3.39 (0.89)
**Overall perceptions of patient safety**	**58.45 (16.54)**	
A15: Patient safety is never sacrificed to get more work done.	39.18	3.19 (0.88)
A18: Our procedures and systems are good at preventing errors form happening.	50.53	2.52(0.85)
A10: It is just by chance that more serious mistakes don’t happen around here.	75	3.93 (0.94)
A17: We have patient safety problems in this unit.	69.07	3.83 (0.70)
**Feedback & communication about error**	**64.72 (7.42)**	
C1: We are given feedback about changes put into place based on event reports.	56.52	3.55 (1.01)
C3: We are informed about errors that happen in this unit.	70.97	3.79 (0.72)
C5: In this unit, we discuss ways to prevent errors form happening again.	66.66	3.76 (0.77)
**Communication openness**	**61.51 (22.74)**	
C2: Staff will freely speak up if they see something that may negatively affect patient care.	69.79	3.70 (0.58)
C4: Staff feel free to question the decisions or actions of those with more authority.	35.79	3.26 (0.76)
C6: Staff are afraid to ask questions when something does not seem right.	78.95	3.93 (0.73)
**Frequency of events reported**	**37.81 (6.90)**	
D1: When a mistake is made, but is caught and corrected before affecting the patient, how often is this reported.	36.9	3.14 (0.93)
D2: When a mistake is made, but has no potential to harm the patient, how often is this reported.	31.4	3.00 (0.98)
D3: When a mistake is made that could harm the patient, but does not, how often is this reported?	45.12	3.37 (0.89)
**Teamwork across units**	**46.88 (12.39)**	
F4: There is good cooperation among hospital units that need to work together.	44.21	3.38 (0.65)
F10: Hospital units work well together to provide the best care for patients.	59.79	3.59 (0.66)
F2: Hospital units do not coordinate well with each other.	30.93	2.99 (0.92)
F6: It is often unpleasant to work with staff from other hospital units.	52.58	3.53 (0.71)
**Staffing**	**62.06 (18.38)**	
A2: We have enough staff to handle the workload.	77.78	3.99 (0.72)
A5: Staff in this unit work longer hours that is best for patient care.	59.26	3.54 (1.03)
A7: We use more agency/temporary staff that is best for patient care.	73.96	3.91 (0.85)
A14: We work in “crisis mode” trying to do too much, too quickly.	37.23	3.27 (0.82)
**Handoffs & transitions**	**47.39 (19.13)**	

F3: Things “fail between the cracks” when transferring patients from one unit to another.	23.47	2.92 (0.8)
F5: Important patient care information is often lost during shift changes.	58.16	3.51(0.76)
F7: Problems often occur in the exchange of information across hospital units.	41.24	3.34 (0.70)
F11: Shift changes are problematic for patients in this hospital.	66.67	3.75 (0.68)
**Nonpunitive response to errors**	**78.73**	
A8: Staff feel like their mistakes are held against them.	76.84	3.91 (0.77)
A12: When an event is reported, t feels like the person is being written up, not the problem.	79.78	3.97 (0.71)
A16: Staff worry that mistakes they make are kept in their personnel file.	79.57	3.98 (0.82)
